# What Are the Stages of the Creative Process? What Visual Art Students Are Saying.

**DOI:** 10.3389/fpsyg.2018.02266

**Published:** 2018-11-21

**Authors:** Marion Botella, Franck Zenasni, Todd Lubart

**Affiliations:** Laboratoire Adaptations Travail-Individu, Université Paris Descartes, Paris, France

**Keywords:** creative process, stages, visual art students, interviews, Creative process Report Diary

## Abstract

Within the literature on creativity in the arts, some authors have focused on the description of the *artistic* process (Patrick, 1937; Getzels and Csikszentmihalyi, 1976; Mace and Ward, 2002; Yokochi and Okada, 2005) whereas others have focused on the *creative* process (Wallas, 1926; Osborn, 1953/1963; Runco and Dow, 1999; Howard et al., 2008). These two types of processes may be, however, somewhat distinct from each other because the creative process is not always dedicated to artistic creation, and productive work in the arts may not always involve creativity, in terms of specifically original thinking. Our goal is to identify the specific nature of the *artistic creative* process, to determine what are the basic stages of this kind of process. This description can then be integrated in a Creative process Report Diary (CRD; Botella et al., 2017) which allows self-observations *in situ* when participants are creating.

## From the existing creative and artistic processes to the artistic creative process

The creative process is defined as a succession of thoughts and actions leading to original and appropriate productions (Lubart, 2001; Lubart et al., 2015). The creative process may be described at two levels: a *macro* level, featuring the stages of the creative process, and a *micro* level, which explains the mechanisms underlying the creative process, e.g., divergent thinking or convergent thinking (Botella et al., 2016). Although the works carried out on micro-processes tend to agree on a set of mechanisms that can be involved in the creative process, work focusing on macroprocesses have not achieved consensus regarding the nature or the number of stages involved in the creative process. Table 1 shows some of the different models that can be found in the scientific literature, with overlaps or divisions between some stages of the models. In this paper, we treat micro-processes as contents of a more global, macro-level process, which make it possible to describe the construction of a work of art from the beginning (i.e., the wish to create) to the end (exhibiting that work). Moreover, the process can be examined in a psychological and individual or in a socio-cultural perspective (Glǎveanu, 2010; Burnard, 2012). In the present study situated in the visual art field, we will consider the artistic creative process as an individual phenomenon.

**Table 1:** Synthesis of some examples of models of creative process.

**Author(s)**								**Stages**							
Wallas, 1926			Preparation					Incubation			Insight				
Guilford, 1956	Sensitivity to problems														
Osborn, 1953/1963		Orientation	Preparation	Analysis				Incubation		Ideation				Synthesis	Evaluation
Busse and Mansfield, 1980		Selection			Efforts	Constraints				Transformation		Verification			
Shaw, 1989, 1994			Immersion					Incubation			Insight	Explanation		Creative synthesis	Validation
Mumford et al., 1994	Problem discovery	Problem definition										Problem construction			
Treffinger, 1995		Understanding								Idea production			Planning		
Amabile, 1988, 1996	Problem presentation		Preparation							Answer generation		Answer validation		Outcome	
Runco, 1997			Information					Incubation			Insight	Verification		Communication	Validation
Doyle, 1998	Incident									Navigation					
Carson, 1999			Preparation		Concentration			Incubation		Ideation	Insight	Verification	Elaboration	Production	
Runco and Dow, 1999	Problem finding							Incubation							Evaluation
Mace and Ward, 2002			Conception							Development				Realization	Finalization
Basadur and Gelade, 2005										Generation		Conceptualization	Optimization	Implementation	
Kilgour, 2006			Definition				Combination			Idea generation					
Howard et al., 2008				Analysis						Generation				Communication/ Implementation	Evaluation
Botella et al., 2011			Preparation		Concentration			Incubation		Ideation	Insight	Verification	Planification	Production	Validation
Cropley and Cropley, 2012			Preparation						Activation	Generation	Insight	Verification			
Botella et al., 2013	Idea or “vision”		Documentation and reflection							First sketches		Testing		Provisional objects		Series
Sadler-Smith, 2016			Preparation					Incubation	Intimation		Insight	Verification			

Art is often considered to be an archetypal domain of creativity research (Schlewitt-Haynes et al., 2002; Stanko-Kaczmarek, 2012), complimented by research on scientific, musical, design-oriented, and literary creativity (Glaveanu et al., 2013). Even if some overlap can be observed between different creative fields, each field has its own specificities (Botella and Lubart, 2015). The purpose of this section is to merge some existing models of the creative process and artistic process to examine what the artistic creative process could be. Obviously, this section cannot be exhaustive but offers a first consideration of the numerous important stages of the artistic creative process.

The process starts by an *orientation*, in which the individual identifies the problem that must be solved (Osborn, 1953/1963), called also a stage of *problem selection* (Busse and Mansfield, 1980) or a *sensitivity to problems* (Guilford, 1956). *Problem definition* involves producing as many questions as possible. For Runco and Dow (1999), *problem-finding* refers to a process of “sensing gaps” (Torrance, 1962)—that is, detecting elements that are lacking. In the same vein, Bruford (2015) proposed a stage of *differentiation* consisting of retaining information that leads to producing something different, involving interpretative and expressive musical differences. Additionally, Mumford et al. (1994) suggested making a distinction between *discovering a problem* (i.e., rejecting problems that are untrue, incorrect, or incomplete; Getzels and Csikszentmihalyi, 1976; Arlin, 1986), *posing the problem* (i.e., finding a correct formulation), and *constructing a problem* (i.e., describing the problem). In the artistic field, Fürst et al. (2012) proposed a model of art production that includes a *goal of creation*.

Then, there is *preparation*, the first stage described in the early macroprocess model by Wallas (1926). Carson (1999) explained that, in this stage, the individual defines the problem (or understands it; Treffinger, 1995) and gathers information in order to solve it. Based on a series of interviews with novelists, Doyle (1998) argued that the creative process begins with an *incident*, when an individual discovers an idea. In the artistic process literature, Mace and Ward (2002) proposed a four-stage model based on interviews with professional artists. For them, the artistic process begins with the *design* of an artistic work. Hence, work is initiated by a more-or-less vague idea or impression. Recently, based also on a series of interviews with professional artists, Botella et al. (2013) identified six stages in the creative process in art, starting by an *idea* or a “*vision*” in which an image, a sight, a sound resonates with the artist.

Before the second main stage described by Wallas (1926), some authors added complementary stages after preparation. Based on a previous review of the literature, Botella et al. (2011) propose a stage of *concentration* (“I am concentrating on the work I have to do”) in which it is possible to focus the creator's attention on those solutions deemed to be adequate, and to reject the other solutions (Carson, 1999). Osborn (1953/1963) added *analysis*, when the creator takes a step back to identify the relations between ideas and the importance of each idea; and *ideation*, when the individual develops alternative ideas. Busse and Mansfield (1980) indicated also a stage requiring making an *effort* in order to solve the problem.

Then, according to Wallas (1926) and many other authors, *incubation* occurs (Osborn, 1953/1963; Shaw, 1989, 1994; Runco, 1997; Runco and Dow, 1999; Botella et al., 2011). This is a time of solitude and relaxation, where idea associations take place at a subconscious level (Carson, 1999). Recently, Sadler-Smith (2016) reintegrated a fifth stage in the Wallas' model: *intimation* occurs between incubation and insight. Intimation is described as an “association-train” in a fringe conscious level, between conscious and unconscious levels (p. 346). Cropley and Cropley (2012) revisited as well Wallas's work and split the stage of incubation into *activation* and *generation*. The process once again becomes conscious in the stage of *ideation*, with the generation of further ideas, which are not necessarily judged or assessed. The individual then experiences an *illumination* or *insight* (Eureka!) with the emergence of an idea, an image or a solution (Wallas, 1926; Carson, 1999). Boden (2004) noted that illumination or insight needs previous thought-processes.

Idea generation can take place in various ways according to the different models. Busse and Mansfield (1980) described a stage in which the creator sets the constraints related to the solution of the problem and, then, another stage involving the transformation of these constraints or adaptation of the constraints that are not suitable. For Doyle (1998), there is some form of *navigation* between various knowledge domains, which makes it possible to assess the relevance of this idea. Based on Dewey (1934), Bruford (2015) proposed a *selection* stage in which the creator choses one option among several, requiring agency and control abilities. In the field of art, Mace and Ward (2002) named this step *idea development* in which the artist structures, completes, and restructures the idea. Botella et al. (2013), through interviews with professional artists identified a stage of *documentation* and *reflection* during which artists gather more information about the materials and technologies required in order to turn their vision into reality. The last stage described by Wallas (1926) is *verification* (Busse and Mansfield, 1980). New ideas are tested and *verified*, leading to the elaboration of a solution and to its production (Carson, 1999). More precisely, Osborn (1953/1963) proposed two distinct phases of *synthesis*, which consists of gathering ideas together and distinguishing relations between them.

Gruber (1989) argued that the four-stage model is incomplete. For Russ (1993), there lacks a stage of application, or deployment of the creative production. Treffinger (1995) added effectively a stage of idea *production*, leading to action by *planning*. This work corresponds to the development and implementation of ideas through a search for solutions (evaluation, selection, and redefinition), and then the acceptance of this solution (promoting an idea, looking for its strengths and drawbacks). This last stage makes it possible to materialize the ideas that have been found and to solve the problem. In this vein, in the field of art, Mace and Ward (2002) described the *realization* of an idea, during which the artist transforms that idea into a physical entity. Botella et al. (2011) also added stages of *planning* (“I am planning my work”), and *production* (“I am producing/composing my ideas”). Results of observations in the art field suggested that the production stage is comprised, in fact, of two stages: a stage that consists of searching for ideas through the creative gesture (sketches, drafts, mock-ups), and then a stage consisting of the realization of an idea that is already constructed (transposing an idea to a concrete medium). The initial stage of “production” describes a similar action, but the underlying cognitive micro-processes are different. In the first case, the goal is to produce in order to formulate an idea whereas in the second case, it is to produce in order to implement an idea that already exists. In a study consisting of interviews of professional artists, Botella et al. (2013) confirmed the stages of first *sketches* to give a material form to the initial project, *testing* the forms and ideas that originated from reflection and preliminary work, and *provisional objects*, “drafts” and almost-finished products. Revisiting Wallas' model, Cropley and Cropley (2012) mentioned a stage of *communication*, as Bruford (2015) with musicians.

For Osborn (1953/1963), the last stage is *evaluation* (Runco and Dow, 1999; or *assessment* for Bruford, 2015), in which the individual assesses the chosen idea. For Mace and Ward (2002), the final step of the artistic process, called *finalization*, brings the artistic work to conclusion (or *validation* according to Botella et al., 2011; Cropley and Cropley, 2012). The artist reassesses the production and may choose to finish, to elaborate, abandon, delay, store, or destroy it. If the artist believes the mission that was set has been accomplished, the artist may choose to exhibit the production. Recently, professional artists suggested to add one more stage with *series*, transforming a first object to many objects (Botella et al., 2013).

All these models were developed based on rational or empirical approaches. Original works and models from Poincaré and Wallas' were conceived based, respectively, on their own experience and pragmatic empirical observations. Patrick (1935, 1937) supported Wallas proposal by collecting empirical data in terms of observations and verbal reports of poets and artists who were invited to do a specific creative task. Most of the “stage models” are then based on this kind of rational or empirical analyses, with verbalizations, specifications, and clarifications of the processes by the participants themselves in the majority of cases. Therefore, these models maybe be considered as a specific approach to creativity, distinct from the psychometric, problem finding or cognitive experimental approaches (Kozbelt et al., 2010). Recent studies on the four-stages model of Wallas confirmed again that researchers do not agree on the number of stages: Cropley and Cropley (2012) found seven stages whereas Sadler-Smith (2016) found five stages based on Wallas' book.

## Objectives

Models of the creative process and of the artistic process do not agree on the nature or on the number of steps involved in a creative artistic process (see Howard et al., 2008). This lack of a consensus could be explained by the fact that (a) the creative process is a complex phenomenon as described by Osborn (1953/1963) who believed that creation is set off by “*stop-and-go*” or “*grab what you can*”-type processes; (b) models of a creative process are constructed based on a specific creative population and a specific creative domain, though these are described as if they were generic and could apply to all domains whether art, science, music, writing, or design. The process is most often described in general terms, as if it should apply to all creative domains, whether it is art, science, music, writing, or design; (c) descriptions of the artistic process do not always take into account the definition of creativity, in particular the contextually rich, situated nature that originality, and appropriateness may have; and (d) the methodologies used were different [be it a review of the literature (Busse and Mansfield, 1980; Botella et al., 2011), a series of interviews with novelists (Doyle, 1998), with professional artists (Mace and Ward, 2002; Botella et al., 2013), or an applied and consulting-based approach (Carson, 1999)].

The aim of the present study is to question directly some stakeholders of artistic creativity, namely visual art students. However, it is maybe too ambitious to ask them to describe completely their creative process. We suggest that the lack of consensus in the previous studies could be due to the desire to capture all aspects of the creative process in the same study. So, the students interviewed here describe only what constitutes, for them, the stages of their process of artistic creativity. We ask them specifically to list the stages of their process in order to be as exhaustive as possible. This qualitative study makes it possible to identify what stages the students consider relevant in their mental representation of the visual artistic creative process, rather than relying on stages extracted from the scientific literature on creativity. With this study, we will not able to have a macro vision of the entire artistic creative process but we will construct an inventory of the stages involved to picture this process.

Given the descriptive nature of the present research on the artistic creative process, the findings can be integrated in further work as a part of the Creative process Report Diary (CRD, Botella et al., 2017). The CRD is a useful and relevant analytical tool to assess the creative process in a natural context, when it occurs, allowing ecological validity. It is possible to realize various versions of the CRD depending on the context, the creative field, and any other considerations. The CRD has two parts: a part listing the stages of the creative process (which will be as exhaustive as possible based on the present study) and a part listing factors such as cognitive, conative, emotional, and environmental ones that may come into the creative process (for example, we could assess team work; Peilloux and Botella, 2016). Finally, the CRD allows the creative process to be modeled for individuals *in situ* during all the time needed for their creation. Thus, the purpose of CRD will be to observe the link and the transitions between the stages of the artistic creative process and to examine which factors will be involved at each stage. However, to do that, we need, in the present study, to list as exhaustively as possible all the stages of the visual artistic creative process which will allow a specific CRD to be created to observe the process in further study.

## Methods

### Participants

The sample was composed of 28 students in the second year of a visual graphic arts school. Seventeen students were female and 11 were male (*mean age* = 20.9 years old, *sd* = 1.7, *span* = 19–24 years old). The rational for the choice of this sample was to interview participants with some artistic experience but to avoid a sample habituated to interviews with strongly formatted ideas. In previous research, when we interviewed professional artists (Botella et al., 2013), we noticed some routines in the discourse. Some artists were familiar with interviews and they narrated a story, usually the story of an artwork but sometimes the reports were distanced from their own story and therefore from their own creative process.

### Interview guide

The goal of the study was to construct a list of the stages of the process of visual artistic creativity. Given this, the interview guide was purposely kept short and open, and consisted of only two questions: (1) “how does your creative process generally take place?” and (2) “how would you name the stages that you have just mentioned?”

The interviewer's follow-up questions allowed the students to describe another stage of their creative process. The main prompts consisted of reformulating the last sentence provided by the participant and asking “When you did […], what do you do next?” or “Can you describe more precisely what you do when you finish […]?” It was very important to not induce ideas with our questions so, we just reformulated the words used by the visual art students themselves to help them list the stages of their artistic creative process.

Interviews were semi-structured and lasted 10 min on average. Obviously, the interviews were too short to capture all the complexity of the artistic creative process with its “stop-and-go” or “grab what you can” aspects (Osborn, 1953/1963). However, to make an inventory of the stages it was enough. The added value of this study is to focus the interview on the stages that visual art students themselves considered and how they named them.

### Procedure

Ethics approval was not required according to our institution's guidelines and national regulations. After the participants provided informed consent, the volunteer students were interviewed in their art school, during their course on creativity. This situation made it easier for them to recall the stages of their visual artistic creative process. Participants were led to a separate room to take part in a one-on-one discussion with the interviewer. The interviewer (and then, the analyst) was the first author, with knowledge on the literature about creativity and creative process, who had already realized many interviews mainly with artists (Botella et al., 2013; Glaveanu et al., 2013). The prompts consisted of reformulating what participants said to assure that we did not induce the use of certain terms.

## Results

Given our objective was to inventory the stages of the artistic creative process, we analyzed the words employed during the interviews. The terms used by students were grouped in equivalence sets using Tropes software which presents references cited at least three times. The name retained for the category was the most cited term; others citations were used to describe the category. In the first part of the analysis, we focus on the stages of the process of visual artistic creativity that emerged spontaneously from the participants' discourse. Hence, we will deal with the responses to the first question in the interview guide. In the second part, we will examine the stages named by the students. Finally, we will confront these two analyses, in order to check whether the stages named by the participants do indeed correspond to those referenced in the discourse. It is expected that the names will be very similar for both analyses but this confrontation serves to cross-check the categorized sets of terms and their labels.

### Identifying the stages of the process from the students' open discourse

Based on the students' responses to the first question in the interview guide, all the terms cited at least three times were listed. It should be noted that the software can already group some terms according to the context: for example, “impossible” and “not possible” are considered as similar. The software can also identify co-occurrences of combined terms, such as “applied art.” Then, terms were grouped by the analyst according to the context in which they appeared (see Table 2). The context helped us to identify the terms concerning the creative process. When terms seem to correspond to the same idea, they were grouped together, such as “Sketchpad,” “sketch,” “drawing,” and “writing.” We conducted an ascendant hierarchical classification, grouping two by two the closest words. The number of clusters was not decided in advance and the grouping was stopped when we considered that another aggregation was not relevant. Terms that did not refer to the creative process were not retained (“year,” “art,” “stage,” “have an inclination toward,” “social environment,” etc.).

**Table 2:** Categories of references used in the students' discourse.

**Category**	**No. times cited**	**Percentage of students (%)**	**Other references used**
Approaching the subject matter	245	100	Apprehending, starting up, word, concept, project, topic, text, theme
Research	203	96.43	Artist, basis, library, searching, cinema, knowledge, culture, curiosity documentation, information, internet, book, research, researching, reference, providing information
Trials	198	92.86	Sketchpad, sketch, drawing, writing, trying, mock-up, note, taking notes
Finalization	119	85.17	Stopping, end, final, finalizing, adding, resuming, correcting, reworking, finishing
Specification	80	85.71	Improving, moving forward, changing, continuing, adding depth, detailing, developing, evolving, perfectionism, photoshop, pushing forward, specifying
Realization	98	75	Composition, make concrete, illustration, implementation, practice, production, clean, production, product, volume
Technique	85	71.43	Code, color, materials, matter, means, painting, photography, style, technique, line, typography, use
Inspiration	51	64.29	Impression, inspiration, inspire, feeling
Reflection	46	57.14	Understanding, deciphering, exploiting, reflecting, reflection, vision, visualization
Organization	38	46.43	Training, link, linking, logic, mixing, order, organizing, steering, connection, relationship
Illumination	37	46.43	Coming across an idea
Presentation	22	42.86	Showing, presentation, handing in
Selection	32	39.29	Choosing, choice, selection, selecting
Judgment	19	35.71	Opinion, looking at
Failure	20	28.57	Failure, mistake, throwing away, missing, beginning again, doing over

*Categories represent the stage of the visual artistic creative process organized by the percent of citation. Number times cited indicates that a student can refer to a category more than one time. All the others references used to mention a stage are reported in this table*.

In Table 2, the number of times that a category was cited and how many students referred to this category are indicated because the same student could mention the same category several times. One stage consists of *approaching* the subject matter, taking possession of it, gaining knowledge about the subject-related words used (S14: “So, you go there, you throw yourself”). *Reflection* refers to the students' efforts for deciphering and understanding the topic. This stage may imply visualized images (S1: “I think, I get things straight for a week”). The stage of *research* involves the student going to the library in order to collect references to artists and to prior work (S4: “I am looking for references to see what has been done. There is a time of documentation”). Then the student constructs a knowledge base of works which have already been produced, before distancing themselves from these works. *Inspiration* is based on one's impression and experience of a given subject matter (S24: “it's really how I feel it and I know I'll be able to continue on it”). Although the term *illumination* was not used, we can note the presence of this stage in students' reports of “an idea suddenly appearing” or “coming across an idea by accident” (S6: “It's not totally conscious. It comes like this. Ideas come alone. We feel it. And after that, we try from that to bring this idea in a frame that could be appropriate”). *Trials* correspond to producing notebooks containing sketches. Students record their sketches, and make attempts before they can find an idea (S27: “I try to explore as many things as possible”). *Organization* consists of students ordering, guiding, and organizing their approach by mixing existing ideas and combining them together (S25: “There is an order to be defined”). The student will have to *select* an idea out of all those produced (S25: “I will select what is best”). A work involves inevitably one or more *techniques* (S18: “Whether computer, photoshop or drawing, rush. Really, exploit everything I know as technical before you get to a final thingy”). Depending on individual preferences and on the constraints of the situation, the student will choose to use a particular technique. The product of the creative process is made concrete during the stage of *realization* (S9: “I go directly to the realization with the materials. I take the painting and I do it directly to clean”). The stage of *specification* indicates that the student improves, specifies and adds the finishing touches to the work (S15: “I am improving what I have already drawn. Above all, I simplify. Because I tend to put too much”). *Finalization* refers to the stage in which the work is completed, finished, and voluntarily stopped (S28: “I am very meticulous and I spend a lot of time on the end”). The stage of *judgment* corresponds to assessing the work that has been produced (S27: “Generally, I have to finish in advance so I can look at it for a long time and then see if something is missing or not. Because sometimes, I have the impression that it is not finished at all and, by dint of looking at it, finally I realize that it misses nothing or that it misses things precisely”). The *presentation* is the moment when students present their work to their teachers (S20: “It's when I show to the teachers”). The stage of *failure* indicates that the student has abandoned something, be it the work or an idea. In the latter case, the student throws away one idea and starts something new, or starts again based on an existing work (S3: “If it's not good, I do not leave, I start again. It happens to me often when I'm done and it's ugly, that I know it's not good, I don't care, I spend another 8 hours, 10 hours to rework another volume. In general, when I resume it's still the same theme, but it's not the same idea”).

### Identifying the stages of the process named by students

This analysis focused on the second question in the interview guide, i.e., how the students named the stages in their visual artistic creative process. Terms were grouped in Table 3. From there, we were able to identify 16 stages in the process of visual artistic creativity.

**Table 3:** Categories used in naming the stages of the creative process.

**Category**	**No. times cited**	**Percentage of students (%)**	**Other references used**
Research	58	78.57	Artist, searching, knowledge, document, information, internet, book, research, researching, reference
Trials	36	64.29	Try-outs, drafts, notebook, croquis, sketch, trial, trying, notes, testing
Realization	27	64.29	Act, action, application, composition, concretization, illustration, implementation, cleaning up, production, transcription, transposition
Selection	12	42.86	Choice, selection
Finalization	12	35.71	Ending, stopping, final, finalization, finalizing, finishing, finishing touches, corrections
Reflection	14	32.14	Brainstorming, understanding, deciphering, reflection, reflecting
Immersion	10	32.14	Assimilation, definition, defining, listening, immersion, impregnation, reception, entering
Specification	10	28.57	Detailing, detail, developing, development, specification
Assembly	9	28.57	Assembly, assembling, setting up ideas
Presentation	7	21.43	Explaining, justifying, speaking, presentation, hand-in
Materials	7	17.86	Photo, volume, materials
Examination	5	17.86	Taking a step back, examining, questioning
Settling	6	14.29	Decanting, digesting, letting time pass, taking a break
Teacher	6	14.29	Teacher
Inspiration	4	14.29	Inspiration, instinct, intuition
Apparition	6	10.71	Apparition, finding, coming
New idea	3	10.71	New, different ideas, differentiation

*Categories represent the stage of the visual artistic creative process organized by the percent of citation. Number times cited indicates that a student can refer to a category more than one time. All the others references used to mention a stage are reported in this table*.

*Immersion* refers to assimilating the work to be done; it involves listening to the instructions given by the teacher, defining the words in the topic, and entering into the project. *Reflection* relates to a form of brainstorming where the student attempts to understand, to decipher the topic and to reflect upon it. *Research* may focus on artists, documents, books, the Internet, and aims for the students to construct a knowledge base for themselves. *Inspiration* seems to be related to intuition and instinct. *Apparition* refers to ideas being found and appearing of their own accord. *Trials* designate all the try-outs, notes, sketches, notes, and testing made by the students. *Assembly* refers both to attempting a new approach and to the different ideas that emerge from assembling ideas together. The stage of *new ideas* includes different ideas which emerge. The stage of *selection* involves choosing an idea. *Materials* were also mentioned in terms of photography and volume. The stage of *realization* refers to action, composition, concretization, production, and to the transfer of an idea to a medium. The stage of *specification* can be viewed as increasing the depth of analysis, developing the work, and correcting it. *Finalization* is the completion of the work. The stage of *examination* indicates taking a step back from the work, formulating an analysis of the work, and questioning one's own work. *Presentation* refers to the fact that students must justify, explain, and present their work. The fact that students let the work *settle*, digest and breathe may refer to the concepts of breaks and incubation. Finally, the *teacher* was also cited as a part of the stages of the process of artistic creativity when students ask for help because they are stuck or when they need reference.

### Confronting the two analyses and identifying the stages in the process of visual artistic creativity

This confrontation allowed us to verify that the students had indeed described all the stages in their creative process, thus validating the number and nature of steps involved in the process to integrate these in the CRD (see Table 4). Fourteen stages appear both in the free discourse and the stages named by the students, one stage was mentioned only in the discourse, and two stages were mentioned when naming the stages of the process. In the end, 17 different stages were retained. Only the stage referring to teacher was not retained because the teacher corresponds more to a social support than a stage of the process. Additionally, the teacher can be partially included in the stage of research as a source of knowledge.

**Table 4:** Confrontation between the two analyses.

**Analysis of the discourse**	**Analysis of the stages named**	**Stages retained**
**Apprehending the subject matter:** Apprehending, starting up, word, concept, project, topic, text, theme	**Immersion:** Assimilation, definition, defining, listening, immersion, impregnation, reception, entering	**Immersion:** Impregnating oneself with the topic, defining the topic, listening to instructions
**Reflection:** Understanding, deciphering, exploiting, reflecting, reflection, vision, visualization	**Reflection:** Brainstorming, understanding, deciphering, reflection, reflecting	**Reflection:** Brainstorming, understanding, visualizing
**Research:** Artist, basis, library, searching, cinema, knowledge, culture, curiosity documentation, information, internet, book, research, researching, reference, providing information	**Research:** Artist, searching, knowledge, document, information, internet, book, research, researching, reference	**Research:** Searching for references, documents, artists, information
**Inspiration:** Impression, inspiration, inspire, feeling	**Inspiration:** Inspiration, instinct, intuition	**Inspiration:** An idea gradually formed, instinct
**Illumination:** Coming across an idea, coming	**Apparition:** Apparition, finding, coming	**Illumination:** “Aha, there's an idea!,” emergence of an idea
**Trials:** Sketchpad, sketch, drawing, writing, trying, mock-up, grading, grade	**Trials:** Try-outs, drafts, notebook, croquis, sketch, trial, trying, notes, testing	**Trials:** Sketches, note-taking, testing, trying out
**Organization:** Training, link, linking, logic, mixing, order, organizing, steering, connection, relationship	**Assembly:** Assembly, assembling, setting up ideas	**Assembly:** Mixing or assembling ideas together
	**New idea:** New, different ideas, differentiation	**Ideation:** Finding and exploring new ideas
**Selection:** Choosing, choice, selection, selecting	**Selection:** Choice, selection	**Selection**: Choosing ideas
**Technique:** Code, color, materials, matter, means, painting, photography, style, technique, line, typography, use	**Materials:** Photography, volume, materials	**Technique:** Choosing a technique, a style, a typography
**Realization:** Composition, make concrete, illustration, implementation, practice, production, clean, product, volume	**Realization:** Act, action, application, composition, concretization, illustration, implementation, cleaning up, production, transcription, transposition	**Realization**: Production, composition, concretization, cleaning up
**Specification:** Improving, moving forward, changing, continuing, adding depth, detailing, developing, evolving, perfectionism, photoshop, pushing forward, specifying	**Specification:** Detailing, detail, developing, development, specification	**Specification:** Refining and detailing
**Finalization:** Stopping, end, final, finalizing, adding, resuming, correcting, reworking, finishing	**Finalization:** Ending, stopping, final, finalization, finalizing, finishing, finishing touches, corrections	**Finalization:** Adding finishing touches, correcting, ending
**Judgment:** Opinion, looking at	**Examination:** Taking a step back, examining, questioning	**Examination:** Taking a step back and examining one's production
**Presentation:** Showing, presentation, handing in	**Presentation:** Explaining, justifying, speaking, presentation, hand-in	**Presentation:** Explaining and justifying one's production
	**Settling:** Decanting, digesting, letting time pass, taking a break	**Break**: Letting everything rest, digesting, decanting
**Failure:** Failure, mistake, throwing away, missing, beginning again, doing over		**Abandoning:** Putting aside or abandoning an idea

In the stage of *immersion*, the goal is to apprehend the topic at hand and to listen to the instructions given by the teacher. Some students may sometimes feel the need to define the words and concepts present in the topic (S1: “What I do personally, I take the words and I take a few days or even a week depending on the time of the project to get things straight, think about it because sometimes there are topics that are very vague like that and we understand not at all. And then it gets more and more precise.”). Such an approach allows them to “soak up” the topic and jump into the fray and start themselves off (S18: “The thing is, I often tend to get into an idea. When you give me a subject or what. I guess right now the thing and what I could do with it.”). *Reflection* makes it possible to understand what should be done, and to decipher the teacher's requirements. Mental work may sometimes begin with visualizing an image. This image may guide the student throughout the process (S20: “Me, I cannot start looking for a word if I do not visualize the final “what.” Even if I will redo after…”). During the stage of *research* the students learn to search for artists, references, documents, and work already produced about the topic that they are apprehending. A solid knowledge base and a culture regarding prior work might help create new and original ideas (S15: “The teachers give us research. Because when we come here, we do not necessarily have a culture in terms of graphics, anyway. They give us references to go see. This is because, often, it is sometimes references of choreographers and it goes a little beyond the field of visual arts and graphics. And suddenly, it allows to compare universes. And then we improve what we do.”). *Inspiration* occurs when an idea emerges slowly and gradually. According to the students, it is based on instinct, impressions, and feelings (S14: “Sometimes you feel that you have a lot of data and from that, you can start to grab something”). Although the word *illumination* was never mentioned, the literature places a strong emphasis on this stage. It is translated in the interviews as “apparition,” “coming across an idea,” and “hey, there's an idea!,” where the idea sometimes comes from an unknown place (S5: “Sometimes it comes alone.”; S21: “I did not look. It fell on me in fact. And so after, you have to bounce back.”). The use of notebooks gather the students' *trials*, their sketches and their notes. They allow the students to try out and test an image. More importantly, the teachers examine the notebooks to follow the evolution of the students' work. Notebooks show students' train of thought, how they achieved a particular work (S2: “These ideas, I always put them in my notebook to show them to the teacher.”). *Assemblies* of ideas are the result of logical connections that the student establishes between several existing ideas. Thus, it corresponds to the direction which the student wishes to give to the production and future work (S3: “I try to mix everything together”). The stage of *ideation* was not mentioned in the discourse. It was only mentioned when students were naming the stages. *Selection* refers to classifying and sorting ideas. The goal here is to choose which ideas can be exploited, and which, on the other hand, should be set aside (S24: “It's hard to choose, on which track to go”). *Technique* is a very important aspect for aspiring artists. They must comply with codes, rules, find a typography, a style of their own. Although this stage was rarely named as such by the students, it is very present in their discourse (S27: “I put in some technique. For example, I had been taught a little about the technique of collage, I had exploited this thing after because I liked it. I tried to distort it from school in my own way.”). *Realization* refers to translating an idea into an image. It is at this point that the composition and production of a material work take shape (S18: “I try to realize it at best”). The stage of *specification* reveals the improvements, the added details, the changes, and corrections made to the work underway. At this point, students add details that they had not necessarily planned initially (S23: “When I have something that I like, I dig it even more to see if I can exploit it”). *Finalization* refers to the point at which the student decides that the work is done. The work is complete, or almost at the point of completion (S17: “It's never finished. For renderings, there is a fixed date and there it is finished. But just for a grade. But in general, we always have stuff to add, photos to resume, stuff to put back. Generally, we do it if we have a jury at the end of the year. And here, we try to finalize the project of the beginning of the year.”). The term *judgment* was not explicitly mentioned either. However, it can be found in the terms of taking a step back, questioning one's work, observing it with great attention, and thus assessing it (S3: “I look at [my work]. I think instead of teachers. If I was a teacher, if I look at, if there is something wrong, if there is a stain, if I see that there is something wrong, if it is not good, well cut, I'll start all over again.”). Although this stage was not directly mentioned in the students' discourse, the stage of the *break* also seems to exist. Its goal is to let the ideas rest, digest, settle and “breathe.” The discourse suggests also the presence of trial and error. Because the word “failure” seems a little strong, we retain the term of “*abandoning*,” whose connotations are less negative (S3: “Sometimes I change my idea and sometimes, when I work, it's not possible like that”).

## Discussion

The goals of this study were to determine the nature and number of stages present in the creative visual artistic process in order to build a specific CRD. Twenty-eight art students were asked to describe their process of visual artistic creativity and to name its stages. By comparing the discourse of these art students and the names they gave to the various stages of their work, we identified 17 stages.

*Immersion* is present in several existing models. It corresponds to preparation in Wallas' (1926) model (see Table 5 for a synthesis). Wallas views preparation as a preliminary analysis which makes it possible to define and set the problem. The same idea is present in Carson's (1999) consulting-centric model and in the work on the creative process of actors (Blunt, 1966; Nemiro, 1997, 1999). Osborn (1953/1963) speaks instead of orientation, in which the individual identifies the problem that is to be solved. Shaw (1989, 1994) proposes also the term “immersion.” *Reflection* is typically included in preparation. Osborn proposes a stage when the individual takes a step back to examine the connections that exist between different ideas. More recently, this stage of reflection was identified in interviews with professional artists (Botella et al., 2013). The stage of *research* is required by the school of art (S8: “We have a lot of instructions from the teachers who help us. We must go through research.”). Research is also generally included in preparation. It should be noted that in Treffinger's model (Treffinger, 1995), preparation is called understanding. The goal here is for the individual to search for information regarding the problem at hand. Also, Runco (1997) mentions a stage of information. Here, the research stage could help visual art students to differentiate their own work from previous ones (Bruford, 2015). In the interviews with professional artists (Botella et al., 2013), this search stage was coupled with reflection, as a search for means (i.e., material or technological) to transform the initial idea into a real production.

**Table 5:** Correspondence between the stages retained in the present study and the existing stages in research field.

**Stages retained in the present study**	**Correspondence with existing stages**
Immersion	*Preparation* (Wallas, 1926; Osborn, 1953/1963; Blunt, 1966; Amabile, 1988; Nemiro, 1997, 1999; Carson, 1999; Botella et al., 2011) *Sensitivity to problems* (Guilford, 1956) *Orientation* (Osborn, 1953/1963) *Immersion* (Shaw, 1989, 1994) *Problem discovery* (Mumford et al., 1994) *Problem presentation* (Amabile, 1988) *Problem finding* (Runco and Dow, 1999) *Conception* (Mace and Ward, 2002) *Definition* (Kilgour, 2006)
Reflection	*Preparation* (Wallas, 1926; Osborn, 1953/1963; Blunt, 1966; Amabile, 1988; Nemiro, 1997, 1999; Carson, 1999; Botella et al., 2011) *Analysis* (Osborn, 1953/1963; Howard et al., 2008) *Efforts* (Busse and Mansfield, 1980) *Problem definition* (Mumford et al., 1994) *Conception* (Mace and Ward, 2002) *Generation* (Basadur and Gelade, 2005; Howard et al., 2008) *Goal of creation* (Fürst et al., 2012) *Documentation* and *reflection* (Botella et al., 2013)
Research	*Preparation* (Wallas, 1926; Osborn, 1953/1963; Blunt, 1966; Amabile, 1988; Nemiro, 1997, 1999; Carson, 1999; Botella et al., 2011) *Efforts* (Busse and Mansfield, 1980) *Problem definition* (Mumford et al., 1994) *Understanding* (Treffinger, 1995) *Information* (Runco, 1997) *Conception* (Mace and Ward, 2002) *Documentation* and *reflection* (Botella et al., 2013) *Differentiation* (Bruford, 2015)
Inspiration	*Intuition* and *metacognition* (Cropley, 1999) *Idea* or *vision* (Botella et al., 2013) *Intimation* (Sadler-Smith, 2016)
Illumination	*Insight* (Wallas, 1926; Ghiselin, 1952; Gruber and Davis, 1988; Weisberg, 1988; Shaw, 1989, 1994; Runco, 1997; Carson, 1999; Botella et al., 2011) *Idea production* (Treffinger, 1995) *Answer generation* (Amabile, 1988) *Incident* (Doyle, 1998) *Conceptualization* (Basadur and Gelade, 2005)
Trials	*Transformation* (Busse and Mansfield, 1980) *Navigation* (Doyle, 1998) *Idea development* (Mace and Ward, 2002) *First sketches* and *testing* (Botella et al., 2013)
Assembly	*Divergent thinking* (Guilford, 1950) *Combination* (Kilgour, 2006)
Ideation	*Ideation* (Osborn, 1953/1963; Carson, 1999; Botella et al., 2011) *Idea generation* (Kilgour, 2006) *Generation* (Cropley and Cropley, 2012)
Selection	*Selection* (Busse and Mansfield, 1980; Bruford, 2015) *Concentration* (Carson, 1999; Botella et al., 2011)
Technique	*Constraints* (Busse and Mansfield, 1980)
Specification	*Elaboration* (Berger et al., 1957; Carson, 1999) *Explanation* (Shaw, 1989, 1994) *Planning* (Treffinger, 1995; Botella et al., 2011) *Optimization* (Basadur and Gelade, 2005) *Documentation* (Botella et al., 2013)
Realization	*Synthesis* (Osborn, 1953/1963; Shaw, 1989, 1994) *Problem construction* (Mumford et al., 1994) *Production* (Treffinger, 1995; Carson, 1999; Botella et al., 2011) *Realization* (Mace and Ward, 2002) *Implementation* (Basadur and Gelade, 2005; Howard et al., 2008) *Provisional objects* (Botella et al., 2013)
Finalization	*Finition* (Mace and Ward, 2002)
Judgement	*Verification* (Wallas, 1926; Busse and Mansfield, 1980; Armbruster, 1989; Runco, 1997; Carson, 1999; Botella et al., 2011) *Evaluation* (Osborn, 1953/1963; Runco and Dow, 1999; Howard et al., 2008) *Validation* (Amabile, 1988; Shaw, 1989, 1994; Runco, 1997; Botella et al., 2011; Cropley and Cropley, 2012) *Assessment* (Bruford, 2015)
Presentation	*Outcome* (Amabile, 1988) *Communication* (Runco, 1997; Howard et al., 2008; Cropley and Cropley, 2012)
Break	*Incubation* (Wallas, 1926; Patrick, 1937; Osborn, 1953/1963; Dreistadt, 1969; Shaw, 1989, 1994; Smith and Blankenship, 1989, 1991; Smith and Vela, 1991; Russ, 1993; Runco, 1997; Carson, 1999; Runco and Dow, 1999; Botella et al., 2011)
Withdrawal	*Abandoning* (Mace and Ward, 2002)

*Inspiration* corresponds to intuition and metacognition (Cropley, 1999). Amongst other things, it allows us to identify which approach will be more efficient than another. Policastro (1995) defines intuition as an implicit form of information processing, which is intended to anticipate and guide creative research. According to her, intuition may allow an unconscious shift from incubation to illumination. However, intuition was never considered a stage in the creative process or in the artistic process. Therefore, it is a stage that is specific to the current study. As described by the students, the inspiration stage is close to the stage on intimation added between incubation and insight (Sadler-Smith, 2016). It is surprising and interesting that visual art students consider inspiration as a stage of their creative process. So, a replication of this study will be necessary to confirm if it is really a stage or if it is a factor involved in the creative process. The word “*illumination*” was not mentioned by the students as such. Numerous authors have previously shown that the illumination stage was seldom mentioned by students in art. Doyle (1998) has described illumination as an accident, where the solution emerges in a sudden and unexpected way (Wallas, 1926). Hence, the description that the students made of this stage might be termed illumination: the idea comes or appears in an unexpected manner. Other authors believe that this experience of illumination would, in most cases, be more gradual than sudden (Ghiselin, 1952; Gruber and Davis, 1988; Weisberg, 1988). Although it is possible that illumination is not a part of all creative processes, or that the creators might not always be conscious of it, the stage of illumination remains a key stage in the creative process, because it is at this stage that the idea takes shape.

The *trials*, tests, and fiddling made by students may correspond to the stage of idea development in Mace and Ward's model (Mace and Ward, 2002). In their description of the artistic process, Mace and Ward argue that, during the development of an idea, the artist will structure, complete, and restructure the idea. Authors indicate that this trial stage will allow artists to form a more precise idea of the initial project for themselves. This stage is worked in Art school with sketchpads.

*Assembly* corresponds to the microprocess of divergent thinking, in which ideas are assembled and mixed together. In contrast, convergent thinking makes it possible to focus on a single idea (Guilford, 1950). This mode of thinking allows individuals to find the one and only solution to a problem. The generation of ideas that have not yet been checked and assessed corresponds to *ideation* (Carson, 1999). Osborn (1953/1963) mentions a stage of synthesis, which consists of putting ideas together and distinguishing relations between them.

*Selection* refers to concentration (Carson, 1999). Concentration makes it possible to focus the attention of the individual on those solutions deemed to be adequate, and to reject other solutions. No model emphasizes the stage of choosing a *technique*. Yet, the artist must identify the technique that will allow them to make the idea materialize in the best possible way. During the interviews with professional artists, technical issues were included in the stage of documentation (Botella et al., 2013). However, in the present study, because 71.43% of the students mentioned this stage in their discourse and 17.86% named it directly, we decided to consider “technique” as a specific stage of the visual artistic creative process. In further studies, it will be interesting to explore if this stage is specific to visual arts or if it is a more common stage concerning other creative domains.

*Specification* might correspond to elaboration. Berger et al. (1957) defined elaboration as the individual's ability to provide detail to the ideas produced. This stage may also tie in with creative explanation, whose goal is for the artist to explain the ideas (Shaw, 1989, 1994).

*Realization* refers to the creative production (Treffinger, 1995) or to creative synthesis (Shaw, 1989, 1994). The goal here is to make the idea concrete. “Technique” is generally included in this stage. However, it seems that production points to the act of creating and to the gestures involved rather than to the cognitive or emotional choice of a technique. Mace and Ward (2002) speak also of realization, i.e., the transformation of an idea into a “physical entity.” They note that for some physical arts and for a wide variety of artistic media it is necessary to have a detailed idea of what the artist is going to do. Hence, some decisions—such as, for example, those related to the choice of a technique—should be anticipated.

*Finalization* corresponds, at least in part, to the finition phase in Mace and Ward (2002). The authors argue that finalization implies that the individual has decided that his/her work is finished. If the artist considers the work to be successful and satisfactory and they may choose to exhibit it. In that case, the stage of finalization also includes hanging up or exhibiting the work.

The stage of *judgement* of the creative production is very often named in models of the creative process. In particular, Wallas (1926) writes about verification, where the individual assesses the idea that has emerged. At this stage, one must take a step back from one's work and assess it. Verification may be of two kinds: “internal” verification, i.e., a comparison between the idea that has been produced and the idea formed during illumination or “internal” verification, which consists of anticipating the reactions of the audience (Armbruster, 1989). According to Busse and Mansfield (1980), verification may take place earlier during the process, as the individual first verifies the ideas and then elaborates a solution. Other authors have argued that judgment occurs at a later stage. For example, Osborn (1953/1963) considers that evaluation is the moment when the individual evaluates the chosen idea. When describing the creative process, Osborn (1953/1963) mentions the stage of analysis, in which the individual takes a step back to examine the connections that form between ideas and their importance. In contrast, Shaw (1989, 1994) addresses the concept of validation, thus emphasizing the importance of this stage. According to him, personal validation consists of appreciating one's own work and in using the experience acquired over the course of this process to generate a new creative process. In addition to personal validation, there exists a collective level of validation. The latter deals with the evaluation of a creative production by peers, by an audience or by a critic. Collective validation can only lead to a new process if there is acceptance of the evaluation that has been formulated. If the production is validated, it can then be followed by a series in which the idea is extended to several works (Botella et al., 2013).

The stage of *presentation* is not typically described as such in models of the creative process or of the artistic process; its goal is to present the work to teachers. In the case of professional artists, this would refer more to the sale of a work. However, recent models included a communication stage (Runco, 1997; Howard et al., 2008; Cropley and Cropley, 2012).

The term “*break*” which has emerged in the stages named by students might correspond to incubation. As we have seen, this stage is very difficult to assess and to take into account (Botella et al., 2011), even though it is essential (Patrick, 1937; Dreistadt, 1969; Smith and Blankenship, 1989, 1991; Smith and Vela, 1991), especially to the expression of artistic creativity (Russ, 1993). The words used by the students highlight some unconscious associations. Indeed, they talk about letting their ideas rest, letting them digest and decant. Incubation is always difficult to evaluate, because it relies in most cases on unconscious work. Finally, although the stage of *withdrawal* is a subject of research, it is not included in most models of the creative process. Only Mace and Ward (2002) take into account a clear possibility of abandoning the process at any time. Even if the process is brutally interrupted, the artist develops continuously new knowledge. This knowledge is the result of a perpetual, dynamic interaction with artistic practice. Artists extend and refine their repertoire of skills, techniques, and knowledge. Also they sharpen their artistic interests and personality. New ideas can emerge in this work, to be reused later.

## Conclusion

Although this study was limited by the interview method—and thus focused on students' implicit theories of their own creative process—it allowed us to identify multiple stages in the process of visual artistic creativity. Because of the implicit theories and the number of models suggesting a linear sequence of stages, sometimes with some loops or cycles possible, it seems too ambitious to understand the sequence of the stages from interviews. The present study invites us to rethink what composes an artistic creative process. Even if we already have a long list of models, none is complete and satisfactory. It is possible that we may need to construct and maintain a list of all the stages of the creative process which can then be adapted to each domain, given that the creative process may vary depending upon the area in question (Baer, 1998, 2010; Botella and Lubart, 2015). Given this uncertainty, continued research into the creative process is indicated. For now, the present list of stages of the visual artistic creative process could help teachers in their coursework. During the interviews, students indicated that the stages of research and the use of the diary notebook were required by their art school. This appears as a limitation of the present study. We are not sure if art students described the prescriptive stages in their Art school or their real stages of creation. The question was oriented how their creative process generally takes place but because they are art students and they were interviewed in their art school, some prescriptive stages appears in their discourse. However, during the interviews, some students had specified if the stage is prescriptive and we indicated this point throughout this paper. With the updated list, teachers could propose other exercises to guide art students for all the stages. Moreover, outside an educational context, the demand for consultancy to stimulate business creativity is increasing (see Berman and Korsten, 2010), and the current research may also provide a helpful template for the effective management of creative processes in this area of industrial innovation. However, we have to be careful about the use of such a list. By conceptualizing the creative process, are we actually at risk of creating a “uniform” prescriptive model of how to be creative? We can hypothesize that some creative process are more adapted to some creative individuals but it would be counterproductive to try to force all individuals to engage in the same process. The creative process varies across fields (Botella and Lubart, 2015) and probably also across culture, creators' personalities, and tasks.

These stages and more precisely their sequence should be validated in the field, by observing students as they carry out artistic work—notably to determine the exact succession of the stages—using a tool like the CRD. Moreover, it will be interesting to observe the collaborative creative process as well as to situate the process in a more global socio-cultural approach. As we saw in the introduction, the creative process can be described using micro-level or macro-level approaches and more globally takes place in a particular socio-cultural context. These approaches could be used directly during observations of the creative process and associated with cognitive, conative, emotional, and environmental factors involved in the process.

## Ethics statement

All subjects gave written informed consent in accordance with the Declaration of Helsinki.

## Author contributions

MB methodology, interviews, analyses, and writing; FZ methodology and writing; and TL methodology and writing.

### Conflict of interest statement

The authors declare that the research was conducted in the absence of any commercial or financial relationships that could be construed as a potential conflict of interest.
